# GLP-1 agonists and exercise: the future of lifestyle prioritization

**DOI:** 10.3389/fcdhc.2025.1720794

**Published:** 2025-11-24

**Authors:** Roberto Codella, Pamela Senesi, Livio Luzi

**Affiliations:** 1 Department of Biomedical Sciences for Health, Università degli Studi di Milano, Milan, Italy; 2 Department of Endocrinology, Nutrition and Metabolic Diseases, Istituto di Ricovero e Cura a Carattere Scientifico (IRCCS) MultiMedica, Milan, Italy

**Keywords:** type 2 diabetes, exercise-as-a-medicine, training, metabolism, health

## Abstract

Recent literature shows that GLP-1 receptor agonists are highly effective for weight loss and improving metabolic and cardiovascular health, often surpassing the results of lifestyle interventions alone, such as exercise and diet modification. However, long-term weight maintenance is more successful when exercise is included, as stopping GLP-1 therapy alone often leads to weight regain, while exercise helps preserve muscle mass and sustain weight loss. Combining GLP-1 receptor agonists with structured lifestyle changes, especially increased protein intake and strength training, can mitigate muscle loss and enhance overall outcomes. As a result, future obesity management is likely to prioritize integrated approaches that combine pharmacotherapy with lifestyle interventions, rather than replacing lifestyle changes with medication alone.

## 1 Introduction

The advent of incretin-based therapies, including glucagon-like peptide-1 receptor agonists (GLP-1RAs) and their combinations, as pharmacological treatments for obesity and type 2 diabetes, has sparked intense debate about their role relative to traditional lifestyle interventions such as exercise and dietary modification. GLP-1 receptor agonists mimic the endogenous incretin hormone GLP-1, exerting multiple physiological effects that contribute to metabolic improvement. They enhance glucose-dependent insulin secretion, suppress glucagon release, slow gastric emptying, and reduce appetite via central hypothalamic pathways, leading to lower energy intake and improved glycemic control. These combined mechanisms underpin their efficacy for both weight reduction and cardiometabolic protection (1, 2). GLP-1RAs, including liraglutide and semaglutide, can induce substantial weight loss (often 10–15% or more), improve cardiometabolic risk factors, including histological damage associated with metabolic dysfunction–associated steatohepatitis (MASH) (3), and reduce major adverse cardiovascular events in both diabetic and non-diabetic populations (1, 4–12).

In recent years, the remarkable efficacy of tirzepatide, a dual GIP/GLP-1 receptor agonist, has emerged (13). While the precise mechanisms of GIP remain incompletely understood in humans, it contributes to energy balance not only by enhancing glucose-dependent insulin secretion but also by modulating adipose tissue metabolism, appetite, and energy expenditure (14). The recent phase 2 trial of tirzepatide in patients with MASH provides support for the therapeutic potential of GIP−pathway modulation in cardiometabolic disease and demonstrates how GIP signaling, when co−targeted with GLP-1RA, may contribute to weight loss, improved metabolic outcomes, and possibly cardiovascular protection (15).

However, these benefits are accompanied by concerns about side effects, cost, long-term sustainability and, above all, the loss of lean mass, which may be mitigated by concurrent exercise (16–20).

Exercise and lifestyle interventions remain foundational for obesity and metabolic disease management, offering broad health benefits, but are often limited by adherence and modest long-term weight loss (11, 16, 18).

Recent randomized controlled trials and meta-analyses suggest that combining GLP-1RAs with exercise may yield additive or synergistic effects on weight loss and metabolic syndrome severity as well as on oxidative stress and inflammation. Moreover, exercise may help maintain weight loss after cessation of pharmacotherapy (16, 17, 19, 21).

The response to these pharmacological treatments is characterized by distinct phases: an initial period of marked weight loss followed by a gradual slowing until a plateau is reached. Some patients may subsequently discontinue therapy due to adverse effects or evolving therapeutic needs. The post-discontinuation phase remains the least investigated, although extension studies consistently show that weight regain occurs rapidly, with patients regaining up to two-thirds of the lost weight within one year after withdrawal (22). Within this dynamic trajectory, structured exercise interventions may play a pivotal role in sustaining weight loss, enhancing metabolic control, and potentially attenuating weight regain after drug discontinuation.

As the field evolves, questions remain about whether the future of obesity and diabetes management will prioritize pharmacotherapy, lifestyle, or a hybrid approach, especially given the challenges of long-term adherence and the broader health impacts of lifestyle change (11, 18, 20, 21).

This narrative review synthesizes current evidence on GLP-1RAs, including both conventional single-agent therapies and dual GIP/GLP-1 receptor agonists, versus exercise, their combined effects, and the implications for future clinical and public health priorities.

## 2 Search strategy and inclusion criteria

This mini-review was based on a targeted literature search conducted in PubMed/MEDLINE and Scopus from January 2015 to August 2025, using the keywords *“GLP-1 receptor agonists,” “exercise,” “physical activity,” “obesity,” “type 2 diabetes,”* and *“weight loss.”* Additional terms (*“lean mass,” “body composition,” “insulin sensitivity,” “cardiometabolic,” “combined intervention”*) were used in secondary searches.

We included peer-reviewed randomized controlled trials, meta-analyses, and high-quality narrative or systematic reviews written in English that evaluated the effects of GLP-1 receptor agonists (liraglutide, semaglutide, or related analogues), as well as dual GIP/GLP-1 receptor agonists (such as tirzepatide), exercise training, or their combination on weight loss, body composition, metabolic, or cardiovascular outcomes in adults with obesity or type 2 diabetes.

Preclinical and mechanistic studies were included selectively when they provided mechanistic insights (e.g., muscle metabolism, neural or hormonal pathways). Grey literature, conference abstracts, and non-peer-reviewed sources were excluded.

The emphasis was on recent and clinically relevant evidence, particularly phase III trials and contemporary meta-analyses that inform translational and hybrid lifestyle–pharmacologic approaches.

## 3 GLP-1 agonists and lifestyle interventions: efficacy, benefits, and limitations for weight control and metabolic health

GLP-1RAs consistently demonstrate significant weight loss (mean reductions of 7–9 kg of body weight over a period of about a year) and improvements in BMI, waist circumference, blood pressure, and lipid profiles in both diabetic and non-diabetic populations (5–7, 10, 23). Cardiovascular outcome trials confirm reductions in major adverse cardiovascular events, and emerging evidence suggests benefits for renal function and inflammation (8, 24–29). However, gastrointestinal side effects and loss of lean mass are notable concerns (5, 10, 17, 18).

Lifestyle interventions, including exercise and dietary modification, remain the cornerstone of obesity and diabetes management, with proven benefits for weight loss, glycemic control, cardiovascular health, and quality of life (11, 16, 18, 30). However, long-term adherence is challenging, and weight loss is often modest and difficult to sustain due to physiological (11, 16, 18, 19). Exercise, particularly resistance training, is crucial for preserving lean mass and functional health, especially during weight loss (11, 17, 18).

### 3.1 GLP-1 agonists and exercise: comparative and combined effects

Direct comparisons reveal that GLP-1RAs generally produce greater short-term weight loss than exercise alone, but exercise is superior for maintaining lean mass and cardiorespiratory fitness (16, 17, 19).

Recent randomized controlled trials show that combining GLP-1RAs with exercise yields additive benefits, including greater reductions in metabolic syndrome severity, abdominal obesity, oxidative stress and inflammation, and improved weight loss maintenance after cessation of pharmacotherapy (16, 17, 19, 21).

Interestingly, recent evidence highlights the interconnection between exercise-induced interleukin-6 (IL-6) secretion and GLP-1RAs. IL-6 release from skeletal muscle has been shown to influence gastric emptying. During physical activity, elevated IL-6 levels can slow gastric motility, contributing to enhanced postprandial satiety and improved glycemic control. This mechanism reflects, in part, the effects of GLP-1RAs, suggesting a convergent pathway through which both exercise and pharmacological therapy modulate gastrointestinal function and metabolic homeostasis.

Hybrid care models integrating pharmacotherapy with lifestyle support show promise for sustainable outcomes (20, 21).

### 3.2 Divergent muscle outcomes in weight loss interventions?

Weight loss interventions based on GLP-1RAs or structured exercise programs both improve share metabolic health, yet their effects on skeletal muscle appear to diverge.

Exercise, particularly when combining aerobic and resistance modalities, exerts well-established benefits on muscle mass, strength, and function (31). These adaptations are mediated by increased mitochondrial biogenesis, enhanced insulin sensitivity, stimulation of anabolic pathways, and the release of myokines such as irisin, apelin, and IL-6, which promote muscle remodeling and systemic metabolic improvements (32).

Preservation of lean mass during weight reduction is crucial, as skeletal muscle is a primary determinant of basal metabolic rate and glucose disposal. Notably, the Physical Activity Working Group of European Association for the Study of Obesity have recently highlighted that resistance training, rather aerobic exercise, attenuates lean body mass loss during weight-loss diets in adults with overweight or obesity (33). Several systemic reviews have confirmed that resistance exercise effectively counteracts potential adverse effects of weight loss by reducing lean mass loss and the associated risk of sarcopenia and frailty (34).

Clinical studies using DEXA show that GLP-1–based weight loss is accompanied by some loss of lean mass, but fat loss predominates. In STEP-1 (semaglutide), lean mass decreased by ~9.7% while fat mass fell by ~19.3%, with the proportion of lean mass increasing by ~3 percentage points (35). In SURMOUNT-1 (tirzepatide), about ~25% of the total weight lost was lean mass and ~75% was fat mass over 72 weeks (36). Real-world liraglutide cohorts report ~22% of total weight loss as lean mass, with an increase in lean-mass percentage (37). Collectively, reviews place the proportion of lean mass in total weight loss at ~15% to 40% (or higher in select settings), reflecting differences in drug, dose, baseline composition, and duration. Regardless of these variations, a central question remains: is the muscle loss associated with GLP-1RAs therapy an adaptive or maladaptive response? (38, 39).

Limited preclinical and clinical data, particularly from MRI-based studies, suggest that skeletal muscle changes induced by GLP-1RAs may be adaptive. Muscle loss appears proportional to aging, disease condition and the degree of weight loss achieved. Emerging research also indicates that GLP-1RAs can directly influence skeletal muscle function. Both liraglutide and semaglutide have been shown to reduce obesity-induced muscle atrophy via a GLP-1/Sirtuin (SIRT1) pathway in rodents (40). Moreover, in obese mice model, semaglutide treatment, despite reducing lean mass, improved skeletal muscle oxidative phosphorylation (OXPHOS) efficiency (41) and ameliorated mitochondrial morphology reducing swelling (42). It is important to emphasize that these findings are very limited and presented in animal and *in vitro* models.

In summary, GLP-1RAs provide substantial weight loss and cardiometabolic benefits but may compromise skeletal muscle integrity, whereas exercise supports muscle maintenance and functional health, though its effect on weight reduction is typically modest compared with pharmacotherapy. A combined approach, where exercise mitigates lean mass loss while GLP-1RAs enhance the magnitude of weight reduction, may represent the most effective therapeutic strategy.

### 3.3 Common neural targets for obesity treatment

Emerging evidence suggests that both GLP-1RAs and physical exercise converge on common neural pathways, influencing brain regions involved in appetite regulation, energy balance, and cognitive function. Exercise induces the release of myokines, such as apelin and irisin, alongside increased levels of BDNF (43, 44). These mediators contribute to central appetite control and neuronal plasticity, while FGF21, especially stimulated by resistance exercise, further modulates hypothalamic circuits and promotes the browning of white adipose tissue, enhancing thermogenesis (45).

GLP-1RAs act on overlapping hypothalamic circuits, reducing appetite and improving energy balance and have been shown to promote browning of white adipose tissue, contributing to increased energy expenditure. McMorrow HE et al. using *in vivo* fiber photometry demonstrated that both GIP and GLP-1 analogs at pharmacologic doses inhibited AgRP neurons in proportion to their anorexigenic action. Notably, dual GIP and GLP-1 receptor agonists produced more potently inhibited AgRP neurons and suppressed food intake than either agonist alone (46). Additional evidence has indicated that GLP-1RAs elevated circulating irisin levels and BDNF (47, 48). As demonstrated by Feetham CH et al., acute activation of BDNF neurons in the medial nucleus of the tractus solitarius reduces food intake and promotes fatty acid oxidation, potentially contributing to sustained weight loss (49).

In line with these finding, Lai S et al. compared the impact of single versus combined treatment with high-intensity interval exercise (HIIE) and GLP-1RA semaglutide on cognitive dysfunction in obese diabetic mice (50). Following eight weeks, both interventions induced significant weight loss and improved glycemic control. Behavioral tests further revealed that semaglutide and HIIE individually enhanced memory and ameliorated depression-like behavior, whereas their combination did not boost cognitive improvements. Mechanistic experiment suggested that both semaglutide and HIIE act through PKA and AMPK signaling pathways, potentially leading to antagonism in regulating BDNF and probably explaining the absence of additive cognitive effects. Similarly, Fontanella RA et al. demonstrated that in neuronal cells that tirzepatide activates Akt/CREB/BDNF pathway and the downstream signaling cascade counteracting hyperglycemia and insulin resistance-related damage at the neuronal level (51). Beyond pharmacological and exercise-bases interventions, novel approaches such as transcranial magnetic stimulation (TMS), have been shown to modulate neuronal activity within circuits regulating feeding behavior and satiety (52).

Altogether, these converging findings highlight the hybrid potential of exercise, GLP-1–based therapies, and neuromodulation to optimize weight management, cognitive function, and metabolic health. Nevertheless, these represent only the first pieces of evidence, and numerous aspects, including the durability of effects, the interplay between signaling pathways, and the potential for synergistic or antagonistic interactions, remain to be clarified by future studies.

### 3.4 Physical activity recommendations in patients with obesity on GLP-1RAs

Patients receiving GLP-1RAs experience significant weight loss but are also at increased risk of lean body mass and bone density loss. Evidence indicates that structured physical activity, particularly resistance training, can mitigate these effects and optimize long-term metabolic outcomes (53–55). A practical, three-step approach – adapted from current World Health Organization (56), American College of Sports Medicine (57), American Diabetes Association (58), and European Association for the Study of Obesity (59) guidelines – is recommended: (1) introduce regular movement gradually, targeting 150 minutes of moderate-intensity or 75 minutes of vigorous aerobic activity per week (60); (2) incorporate resistance training for 60–90 minutes weekly, using accessible methods such as resistance bands, weights, or bodyweight exercises (55, 60, 61); and (3) sustain long-term engagement with 30–60 minutes of daily aerobic activity alongside resistance training 2–3 times weekly (61) (**Table 1**). Exercise prescriptions should be individualized and progressively adjusted according to age, comorbidities, baseline fitness, and tolerance to weight loss, with attention to injury prevention and recovery. Functional assessments (e.g., grip strength, 6-minute walk test) can help monitor progress. Integrating these exercise strategies with nutritional support is essential to preserve muscle mass, reduce fatigue, and maximize the benefits of GLP-1RA therapy (62).

**Table 1:** Physical activity recommendations in people with obesity on GLP-1RAs.

Step	Type of activity	Frequency & duration	Goal/benefit
1. Gradual Movement	Brisk walking, light aerobic activity	Start with 10 min/day → build to 150 min/wk (moderate) or 75 min/wk (vigorous)	Improves aerobic fitness, reduces fatigue
2. Resistance Training	Bands, weights, or bodyweight (squats, lunges)	2–3 sessions/wk, 20–30 min each (total 60–90 min/wk)	Preserves lean body mass, bone density, muscle strength
3. Maintenance	Combined aerobic + resistance training	30–60 min aerobic daily + 2–3 resistance sessions/wk	Optimizes long-term weight control, metabolic health, and function
Additional focus	Balance and mobility	Integrated into weekly routine, especially for older adults	Prevents sarcopenia and reduces fall risk

## 4 Discussion

Current evidence strongly supports the efficacy of GLP-1 receptor agonists for inducing substantial weight loss, improving metabolic control, and reducing cardiovascular risk – often surpassing the short-term effects of exercise alone (4–10, 23). However, these pharmacologic benefits are tempered by important trade-offs, including gastrointestinal side effects, treatment cost, and loss of lean mass, which may have long-term functional implications if not counteracted by concurrent resistance exercise (17–19). In fact, GLP-1RAs reduce appetite and gastric emptying – mechanisms that, while beneficial for weight loss, may also limit protein intake and nutrient absorption necessary for muscle preservation.

Exercise and lifestyle interventions, while generally less potent for weight loss, provide unique and irreplaceable health benefits – preserving skeletal muscle, improving cardiorespiratory fitness, enhancing psychological well-being, and lowering chronic disease risk (11, 16, 18, 19). Recent trials indicate that combining GLP-1RAs with structured exercise programs yields additive or even synergistic effects – enhancing weight loss, preserving muscle mass, and supporting long-term metabolic stability (16, 19, 21).

Hybrid care models that integrate pharmacotherapy with behavioral support are emerging as the most promising framework for sustainable obesity and diabetes management (20, 21). Yet, enthusiasm for GLP-1RAs should not eclipse the foundational role of lifestyle modification, which remains essential for holistic health and may mitigate some pharmacotherapy risks (11, 18, 20).

Persistent challenges include the high cost and limited accessibility of GLP-1RAs, variable long-term adherence, and a lack of long-term data on sustainability, relapse rates, and overall health outcomes. Furthermore, direct comparisons of GLP-1RAs and exercise in diverse populations are scarce, and evidence on optimal sequencing, duration, and integration of hybrid interventions remains limited (1, 18).

Beyond physiological adaptations, treatment adherence and long-term success largely depend on motivation, self-efficacy, and body-image perception, which may fluctuate during pharmacologic weight loss (63). Rapid weight reduction induced by GLP-1RAs can improve body satisfaction and quality of life, but it may also create unrealistic expectations or dependence on medication for weight control (64). Conversely, structured exercise programs foster intrinsic motivation (65), self-regulation, and a positive relationship with the body, promoting sustained engagement even after pharmacotherapy discontinuation. However, adherence to physical activity remains a major challenge, often limited by perceived effort, time constraints, and lack of behavioral support. Integrating behavioral counseling and psychological monitoring – for instance through motivational interviewing, cognitive-behavioral strategies, or digital adherence tools – could enhance both pharmacologic and lifestyle outcomes.

Future research should focus on refining hybrid strategies, optimizing dose-exercise combinations, and addressing health equity and implementation barriers. Policymakers and clinicians alike must ensure that effective, evidence-based treatments – pharmacologic and behavioral – are accessible and sustainable across populations.

### 4.1 Limitations of the current literature

Although research examining the combined and comparative effects of GLP-1 receptor agonists and exercise is rapidly expanding, several limitations constrain the current evidence base. First, there is a lack of standardized exercise protocols across studies, with wide variability in training type, frequency, intensity, and supervision, which limits comparability and prevents the identification of optimal regimens for preserving lean mass or enhancing metabolic outcomes. Second, most available trials are characterized by short follow-up periods (generally ≤6–12 months), providing limited insight into the durability of weight loss, muscle preservation, or cardiometabolic benefits after treatment cessation. Furthermore, reporting heterogeneity – including diverse measures of body composition and inconsistent endpoints – hampers cross-study synthesis. Finally, a publication bias toward studies reporting positive or synergistic effects between GLP-1RAs and exercise may inflate perceived efficacy. Future investigations should implement standardized exercise prescriptions, longer follow-up, and preregistered multicenter designs to ensure methodological consistency and improve translational relevance.

## 5 Conclusion

**Figure 1:**
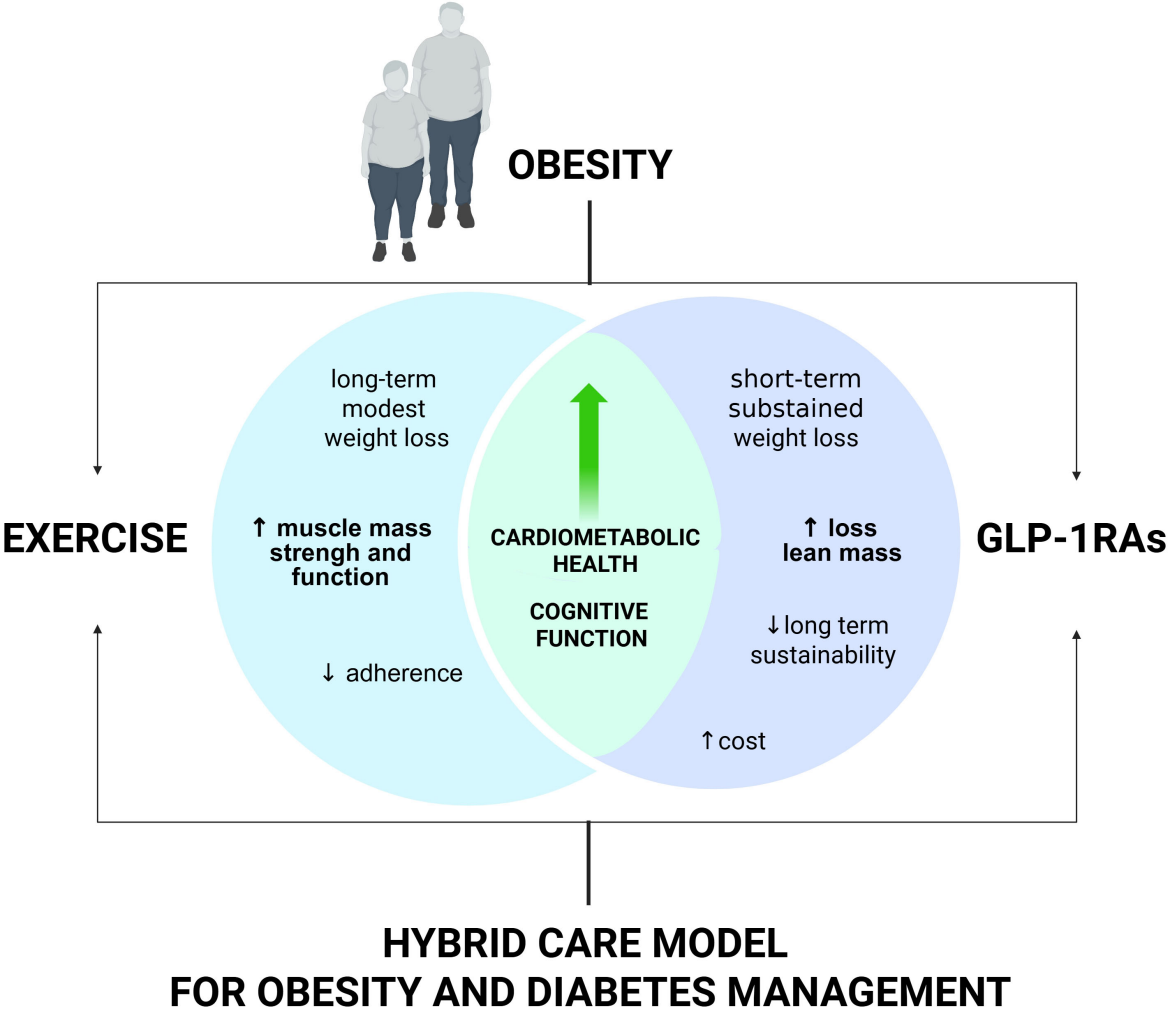
Combined effects of GLP-1RAs and exercise on weight and muscle health. GLP-1Ras promote significant weight loss and cardiometabolic benefits but may reduce skeletal muscle integrity. Exercise supports muscle maintenance and function, though its impact on weight is modest. A combined approach may maximize weight reduction while preserving lean mass.

In summary, GLP-1RAs have revolutionized obesity and diabetes management, offering potent weight loss and cardiometabolic benefits, but are best used in conjunction with lifestyle interventions, particularly exercise, to optimize health outcomes and sustainability. The future will likely prioritize hybrid models that integrate pharmacotherapy with structured lifestyle support, but further research is needed to address long-term maintenance, cost, and health equity (**Figure 1**).

## References

[1] Holst JJ . GLP-1 physiology in obesity and development of incretin-based drugs for chronic weight management. Nat Metab. (2024) 6:1866–85. doi: 10.1038/s42255-024-01113-9, PMID: 39160334

[2] Drucker DJ . Mechanisms of action and therapeutic application of glucagon-like peptide-1. Cell Metab. (2018) 27:740–56. doi: 10.1016/j.cmet.2018.03.001, PMID: 29617641

[3] Sanyal AJ Newsome PN Kliers I Østergaard LH Long MT Kjær MS . Phase 3 trial of semaglutide in metabolic dysfunction-associated steatohepatitis. N Engl J Med. (2025) 392:2089–99. doi: 10.1056/NEJMoa2413258, PMID: 40305708

[4] Wang J-Y Wang Q-W Yang X-Y Yang W Li D-R Jin J-Y . GLP–1 receptor agonists for the treatment of obesity: Role as a promising approach. Front Endocrinol (Lausanne). (2023) 14:1085799. doi: 10.3389/fendo.2023.1085799, PMID: 36843578 PMC9945324

[5] Ansari HUH Qazi SU Sajid F Altaf Z Ghazanfar S Naveed N . Efficacy and safety of glucagon-like peptide-1 receptor agonists on body weight and cardiometabolic parameters in individuals with obesity and without diabetes: A systematic review and meta-analysis. Endocrine Pract. (2024) 30:160–71. doi: 10.1016/j.eprac.2023.11.007, PMID: 38029929

[6] Wong HJ Sim B Teo YH Teo YN Chan MY Yeo LLL . Efficacy of GLP-1 receptor agonists on weight loss, BMI, and waist circumference for patients with obesity or overweight: A systematic review, meta-analysis, and meta-regression of 47 randomized controlled trials. Diabetes Care. (2025) 48:292–300. doi: 10.2337/dc24-1678, PMID: 39841962

[7] Popoviciu M-S Păduraru L Yahya G Metwally K Cavalu S . Emerging role of GLP-1 agonists in obesity: A comprehensive review of randomised controlled trials. Int J Mol Sci. (2023) 24:10449. doi: 10.3390/ijms241310449, PMID: 37445623 PMC10341852

[8] Ussher JR Drucker DJ . Glucagon-like peptide 1 receptor agonists: cardiovascular benefits and mechanisms of action. Nat Rev Cardiol. (2023) 20:463–74. doi: 10.1038/s41569-023-00849-3, PMID: 36977782

[9] Baggio LL Drucker DJ . Glucagon-like peptide-1 receptor co-agonists for treating metabolic disease. Mol Metab. (2021) 46:101090. doi: 10.1016/j.molmet.2020.101090, PMID: 32987188 PMC8085566

[10] Iqbal J Wu H Hu N Zhou Y Li L Xiao F . Effect of glucagon-like peptide-1 receptor agonists on body weight in adults with obesity without diabetes mellitus—a systematic review and meta-analysis of randomized control trials. Obes Rev. (2022) 23(6):e13435. doi: 10.1111/obr.13435, PMID: 35194917

[11] Davies MJ D’Alessio DA Fradkin J Kernan WN Mathieu C Mingrone G . Management of hyperglycemia in type 2 diabetes, 2018. A consensus report by the american diabetes association (ADA) and the european association for the study of diabetes (EASD). Diabetes Care. (2018) 41:2669–701. doi: 10.2337/dci18-0033, PMID: 30291106 PMC6245208

[12] Drucker DJ . GLP-1 physiology informs the pharmacotherapy of obesity. Mol Metab. (2022) 57:101351. doi: 10.1016/j.molmet.2021.101351, PMID: 34626851 PMC8859548

[13] Wen J Syed B Nadora D How-Volkman C Bernstein E Truong A . Tirzepatide versus semaglutide on weight loss in type 2 diabetes patients: A systematic review and meta-analysis of direct comparative studies. Endocrinol Diabetes Metab. (2025) 8:e70045. doi: 10.1002/edm2.70045, PMID: 40184508 PMC11970626

[14] Yamane S Harada N Inagaki N . Physiology and clinical applications of GIP. Endocr J. (2025) 72:751–64. doi: 10.1507/endocrj.EJ25-0087, PMID: 40175127 PMC12260194

[15] Loomba R Hartman ML Lawitz EJ Vuppalanchi R Boursier J Bugianesi E . Tirzepatide for metabolic dysfunction-associated steatohepatitis with liver fibrosis. N Engl J Med. (2024) 391:299–310. doi: 10.1056/NEJMoa2401943, PMID: 38856224

[16] Sandsdal RM Juhl CR Jensen SBK Lundgren JR Janus C Blond MB . Combination of exercise and GLP-1 receptor agonist treatment reduces severity of metabolic syndrome, abdominal obesity, and inflammation: a randomized controlled trial. Cardiovasc Diabetol. (2023) 22:41. doi: 10.1186/s12933-023-01765-z, PMID: 36841762 PMC9960425

[17] Locatelli JC Costa JG Haynes A Naylor LH Fegan PG Yeap BB . Incretin-based weight loss pharmacotherapy: can resistance exercise optimize changes in body composition? Diabetes Care. (2024) 47:1718–30. doi: 10.2337/dci23-0100, PMID: 38687506

[18] Dash S . Opportunities to optimize lifestyle interventions in combination with glucagon-like peptide -1-based therapy. Diabetes Obes Metab. (2024) 26:3–15. doi: 10.1111/dom.15829, PMID: 39157881

[19] Jensen SBK Blond MB Sandsdal RM Olsen LM Juhl CR Lundgren JR . Healthy weight loss maintenance with exercise, GLP-1 receptor agonist, or both combined followed by one year without treatment: a post-treatment analysis of a randomised placebo-controlled trial. EClinicalMedicine. (2024) 69:102475. doi: 10.1016/j.eclinm.2024.102475, PMID: 38544798 PMC10965408

[20] Mozaffarian D . GLP-1 agonists for obesity—A new recipe for success? JAMA. (2024) 331:1007. doi: 10.1001/jama.2024.2252, PMID: 38421659

[21] Zakaria H Alshehhi S Caccelli M Ozkan C Kattan J Jafaar Z . Effectiveness of a hybrid approach in integrating GLP-1 agonists and lifestyle guidance for obesity and pre-diabetes management: RWE retrospective study. Metabol Open. (2024) 22:100283. doi: 10.1016/j.metop.2024.100283, PMID: 38699398 PMC11064605

[22] Wilding JPH Batterham RL Davies M Van Gaal LF Kandler K Konakli K . Weight regain and cardiometabolic effects after withdrawal of semaglutide: The STEP 1 trial extension. Diabetes Obes Metab. (2022) 24:1553–64. doi: 10.1111/dom.14725, PMID: 35441470 PMC9542252

[23] Moll H Frey E Gerber P Geidl B Kaufmann M Braun J . GLP-1 receptor agonists for weight reduction in people living with obesity but without diabetes: a living benefit–harm modelling study. EClinicalMedicine. (2024) 73:102661. doi: 10.1016/j.eclinm.2024.102661, PMID: 38846069 PMC11154119

[24] Alexiadou K Hartley A Tan TM-M Khamis R . The cardiovascular effects of GLP-1 receptor agonists beyond obesity and type 2 diabetes: An anti-atherosclerotic action. Trends Cardiovasc Med. (2024) 34:552–7. doi: 10.1016/j.tcm.2024.03.003, PMID: 38555029

[25] Pedrosa MR Franco DR Gieremek HW Vidal CM Bronzeri F de Cassia Rocha A . GLP-1 agonist to treat obesity and prevent cardiovascular disease: what have we achieved so far? Curr Atheroscler Rep. (2022) 24:867–84. doi: 10.1007/s11883-022-01062-2, PMID: 36044100

[26] Ma X Liu Z Ilyas I Little PJ Kamato D Sahebka A . GLP-1 receptor agonists (GLP-1RAs): cardiovascular actions and therapeutic potential. Int J Biol Sci. (2021) 17:2050–68. doi: 10.7150/ijbs.59965, PMID: 34131405 PMC8193264

[27] Zhang X Cao C Zheng F Liu C Tian X . Therapeutic potential of GLP-1 receptor agonists in diabetes and cardiovascular disease: mechanisms and clinical implications. Cardiovasc Drugs Ther. (2025). doi: 10.1007/s10557-025-07670-9, PMID: 39832069 PMC12872670

[28] Bendotti G Montefusco L Lunati ME Usuelli V Pastore I Lazzaroni E . The anti-inflammatory and immunological properties of GLP-1 Receptor Agonists. Pharmacol Res. (2022) 182:106320. doi: 10.1016/j.phrs.2022.106320, PMID: 35738455

[29] Le R Nguyen MT Allahwala MA Psaltis JP Marathe CS Marathe JA . Cardiovascular protective properties of GLP-1 receptor agonists: more than just diabetic and weight loss drugs. J Clin Med. (2024) 13:4674. doi: 10.3390/jcm13164674, PMID: 39200816 PMC11355214

[30] Yun J-S Ko S-H . Current trends in epidemiology of cardiovascular disease and cardiovascular risk management in type 2 diabetes. Metabolism. (2021) 123:154838. doi: 10.1016/j.metabol.2021.154838, PMID: 34333002

[31] Oppert J-M Ciangura C Bellicha A . Physical activity and exercise for weight loss and maintenance in people living with obesity. Rev Endocr Metab Disord. (2023) 24:937–49. doi: 10.1007/s11154-023-09805-5, PMID: 37142892

[32] van Baak MA Pramono A Battista F Beaulieu K Blundell JE Busetto L . Effect of different types of regular exercise on physical fitness in adults with overweight or obesity: Systematic review and meta-analyses. Obes Rev. (2021) 22(Suppl 4):e13239. doi: 10.1111/obr.13239, PMID: 33939229 PMC8365680

[33] Oppert J Bellicha A van Baak MA Battista F Beaulieu K Blundell JE . Exercise training in the management of overweight and obesity in adults: Synthesis of the evidence and recommendations from the European Association for the Study of Obesity Physical Activity Working Group. Obes Rev. (2021) 22(Suppl 4):e13273. doi: 10.1111/obr.13273, PMID: 34076949 PMC8365734

[34] Lopez P Taaffe DR Galvão DA Newton RU Nonemacher ER Wendt VM . Resistance training effectiveness on body composition and body weight outcomes in individuals with overweight and obesity across the lifespan: A systematic review and meta-analysis. Obes Rev. (2022) 23(5):e13428. doi: 10.1111/obr.13428, PMID: 35191588 PMC9285060

[35] Wilding JPH Batterham RL Calanna S Davies M Van Gaal LF Lingvay I . Once-weekly semaglutide in adults with overweight or obesity. New Engl J Med. (2021) 384:989–1002. doi: 10.1056/NEJMoa2032183, PMID: 33567185

[36] Look M Dunn JP Kushner RF Cao D Harris C Gibble TH . Body composition changes during weight reduction with tirzepatide in the SURMOUNT -1 study of adults with obesity or overweight. Diabetes Obes Metab. (2025) 27:2720–9. doi: 10.1111/dom.16275, PMID: 39996356 PMC11965027

[37] Santini S Vionnet N Pasquier J Gonzalez-Rodriguez E Fraga M Pitteloud N . Marked weight loss on liraglutide 3.0 mg: Real-life experience of a S wiss cohort with obesity. Obesity. (2023) 31:74–82. doi: 10.1002/oby.23596, PMID: 36478514 PMC10107497

[38] Neeland IJ Linge J Birkenfeld AL . Changes in lean body mass with glucagon-like peptide -1-based therapies and mitigation strategies. Diabetes Obes Metab. (2024) 26:16–27. doi: 10.1111/dom.15728, PMID: 38937282

[39] Linge J Birkenfeld AL Neeland IJ . Muscle mass and glucagon-like peptide-1 receptor agonists: adaptive or maladaptive response to weight loss? Circulation. (2024) 150:1288–98. doi: 10.1161/CIRCULATIONAHA.124.067676, PMID: 39401279

[40] Xiang J Qin L Zhong J Xia N Liang Y . GLP-1RA liraglutide and semaglutide improves obesity-induced muscle atrophy via SIRT1 pathway. Diabetes Metab Syndrome Obes. (2023) 16:2433–46. doi: 10.2147/DMSO.S425642, PMID: 37602204 PMC10439806

[41] Choi RH Karasawa T Meza CA Maschek JA Manuel AM Nikolova LS . Semaglutide-induced weight loss improves mitochondrial energy efficiency in skeletal muscle. Obesity. (2025) 33:974–85. doi: 10.1002/oby.24274, PMID: 40254778 PMC12015655

[42] Old VJ Davies MJ Papamargaritis D Choudhary P Watson EL . The effects of glucagon-like peptide-1 receptor agonists on mitochondrial function within skeletal muscle: A systematic review. J Cachexia Sarcopenia Muscle. (2025) 16(1):e13677. doi: 10.1002/jcsm.13677, PMID: 39815782 PMC11735953

[43] Bian X Wang Q Wang Y Lou S . The function of previously unappreciated exerkines secreted by muscle in regulation of neurodegenerative diseases. Front Mol Neurosci. (2024) 16:1305208. doi: 10.3389/fnmol.2023.1305208, PMID: 38249295 PMC10796786

[44] Wei M Wu T Chen N . Bridging neurotrophic factors and bioactive peptides to Alzheimer’s disease. Ageing Res Rev. (2024) 94:102177. doi: 10.1016/j.arr.2023.102177, PMID: 38142891

[45] Liu C Yan X Zong Y He Y Yang G Xiao Y . The effects of exercise on FGF21 in adults: a systematic review and meta-analysis. PeerJ. (2024) 12:e17615. doi: 10.7717/peerj.17615, PMID: 38948228 PMC11212618

[46] McMorrow HE Cohen AB Lorch CM Hayes NW Fleps SW Frydman JA . Incretin receptor agonism rapidly inhibits AgRP neurons to suppress food intake in mice. J Clin Invest. (2025) 135(21):e186652. doi: 10.1172/JCI186652, PMID: 40857106 PMC12578400

[47] Guarnotta V Bianco MJ Vigneri E Panto’ F Lo Sasso B Ciaccio M . Effects of GLP-1 receptor agonists on myokine levels and pro-inflammatory cytokines in patients with type 2 diabetes mellitus. Nutr Metab Cardiovasc Dis. (2021) 31:3193–201. doi: 10.1016/j.numecd.2021.07.015, PMID: 34518091

[48] Marrano N Biondi G Borrelli A Cignarelli A Perrini S Laviola L . Irisin and incretin hormones: similarities, differences, and implications in type 2 diabetes and obesity. Biomolecules. (2021) 11:286. doi: 10.3390/biom11020286, PMID: 33671882 PMC7918991

[49] Feetham CH Collabolletta V Worth AA Shoop R Groom S Harding C . Brainstem BDNF neurons are downstream of GFRAL/GLP1R signalling. Nat Commun. (2024) 15:10749. doi: 10.1038/s41467-024-54367-y, PMID: 39737892 PMC11685588

[50] Lai S Kang Z Sun J Wang Z Xu Y Xing S . Semaglutide and high-intensity interval exercise attenuate cognitive impairment in type 2 diabetic mice via BDNF modulation. Brain Sci. (2025) 15(5):480. doi: 10.3390/brainsci15050480, PMID: 40426650 PMC12109977

[51] Fontanella RA Ghosh P Pesapane A Taktaz F Puocci A Franzese M . Tirzepatide prevents neurodegeneration through multiple molecular pathways. J Transl Med. (2024) 22:114. doi: 10.1186/s12967-024-04927-z, PMID: 38287296 PMC10823712

[52] Ferrulli A Macrì C Terruzzi I Massarini S Ambrogi F Adamo M . Weight loss induced by deep transcranial magnetic stimulation in obesity: A randomized, double-blind, sham-controlled study. Diabetes Obes Metab. (2019) 21:1849–60. doi: 10.1111/dom.13741, PMID: 30957981

[53] Mehrtash F Dushay J Manson JE . Integrating diet and physical activity when prescribing GLP-1s—Lifestyle factors remain crucial. JAMA Intern Med. (2025) 185:1151. doi: 10.1001/jamainternmed.2025.1794, PMID: 40658434

[54] Mehrtash F Dushay J Manson JE . I am taking a GLP-1 weight-loss medication—What should I know? JAMA Intern Med. (2025) 185:1180. doi: 10.1001/jamainternmed.2025.1133, PMID: 40658429

[55] Bagherzadeh-Rahmani B Marzetti E Karami E Campbell BI Fakourian A Haghighi AH . Tirzepatide and exercise training in obesity. Clin Hemorheol Microcirc. (2024) 87:465–80. doi: 10.3233/CH-242134, PMID: 38640145

[56] Bull FC Al-Ansari SS Biddle S Borodulin K Buman MP Cardon G . World Health Organization 2020 guidelines on physical activity and sedentary behaviour. Br J Sports Med. (2020). 54(24):1451–62. doi: 10.1136/bjsports-2020-102955, PMID: 33239350 PMC7719906

[57] Ozemek C Bonikowske AR Christle JW Gallo PM . ACSM’s guidelines for exercise testing and prescription. Cemal, Ozemak: Wolters Kluwer (2026). 645 p.

[58] ElSayed NA Aleppo G Bannuru RR Bruemmer D Collins BS Ekhlaspour L . 2. Diagnosis and classification of diabetes: *standards of care in diabetes—2024*. Diabetes Care. (2024) 47:S20–42. doi: 10.2337/dc24-S002, PMID: 38078589 PMC10725812

[59] Müller MJ . Reports of the EASO physical activity working group: Diverse insights, evidence-based recommendations, and future perspectives. Obes Rev. (2021) 22(Suppl 4):e13254. doi: 10.1111/obr.13254, PMID: 33855797 PMC8365737

[60] Wadden TA Chao AM Moore M Tronieri JS Gilden A Amaro A . The role of lifestyle modification with second-generation anti-obesity medications: comparisons, questions, and clinical opportunities. Curr Obes Rep. (2023) 12:453–73. doi: 10.1007/s13679-023-00534-z, PMID: 38041774 PMC10748770

[61] Colberg SR Sigal RJ Yardley JE Riddell MC Dunstan DW Dempsey PC . Physical activity/exercise and diabetes: A position statement of the american diabetes association. Diabetes Care. (2016) 39:2065–79. doi: 10.2337/dc16-1728, PMID: 27926890 PMC6908414

[62] Almandoz JP Wadden TA Tewksbury C Apovian CM Fitch A Ard JD . Nutritional considerations with antiobesity medications. Obesity. (2024) 32:1613–31. doi: 10.1002/oby.24067, PMID: 38853526

[63] Arillotta D Floresta G Guirguis A Corkery JM Catalani V Martinotti G . GLP-1 receptor agonists and related mental health issues; insights from a range of social media platforms using a mixed-methods approach. Brain Sci. (2023) 13:1503. doi: 10.3390/brainsci13111503, PMID: 38002464 PMC10669484

[64] Gleason PP Urick BY Marshall LZ Friedlander N Qiu Y Leslie RS . Real-world persistence and adherence to glucagon-like peptide-1 receptor agonists among obese commercially insured adults without diabetes. J Manag Care Spec Pharm. (2024) 30:860–7. doi: 10.18553/jmcp.2024.23332, PMID: 38717042 PMC11293763

[65] Ryan RM Williams GC Patrick H Deci EL . Self-determination theory and physical activity: The dynamics of motivation in development and wellness. Hellenic J Psychol. (2009) 6:107–24. doi: 10.1186/1479-5868-9-78, PMID: 22726453 PMC3441783

