# Supplementary material for: GLP-1 agonists and exercise: the future of lifestyle prioritization
Source: Front Clin Diabetes Healthc. 2025 Nov 24;6:1720794. doi: 10.3389/fcdhc.2025.1720794 (PMC12683586; doi:10.3389/fcdhc.2025.1720794)
Supplement: Supplementary file 1 [file Table1.docx]

| **Drug (Trial)** | **Modality** | **Duration** | **Body Weight Change** | **Fat Mass Change** | **Lean Mass Change** | **Notes / Key Findings** |
| --- | --- | --- | --- | --- | --- | --- |
| Semaglutide (STEP-1, Wilding et al., 2021) | GLP-1RA 2.4 mg weekly vs placebo + lifestyle | 68 weeks | −14.9% vs −2.4% | −19.3% | −9.7% (≈35–40% of total weight loss) | Fat loss predominates; lean mass % increased by ~3 points; improved cardiometabolic markers |
| Tirzepatide (SURMOUNT-1, Jastreboff et al., 2022) | Dual GIP/GLP-1RA vs placebo | 72 weeks | −15–21% (dose-dependent) | ~−25% fat mass | ~−5–6% (≈25% of total weight loss) | Greater weight reduction vs semaglutide; improved insulin sensitivity |
| Liraglutide (SCALE, Pi-Sunyer et al., 2015) | GLP-1RA 3.0 mg daily vs placebo + lifestyle | 56 weeks | −8.0% vs −2.6% | −12–15% | −3–4% (≈20–25% of total loss) | GI side effects common; improved cardiometabolic risk profile |
| Semaglutide (STEP-4, Rubino et al., 2021) | Continuation vs withdrawal phase | 68 + 48 weeks | Weight regain of ~2/3 within 1 yr after withdrawal | – | – | Highlights need for maintenance strategies (exercise, diet) post-therapy |
| GLP-1RA + Exercise (Kawaguchi et al., 2022) | Liraglutide + aerobic/resistance training | 24 weeks | −7.4% vs −3.2% (exercise only) | – | Lean mass better preserved | Additive metabolic benefits, reduced inflammation |
| GLP-1RA + Exercise (Krause et al., 2023) | Semaglutide + supervised aerobic training | 20 weeks | −12.5% vs −9.8% (drug only) | ↓ visceral fat | ↓ lean loss vs drug alone | Additive benefit on fitness, lipid profile, oxidative stress |
| Tirzepatide + Lifestyle (Frías et al., 2021) | GIP/GLP-1 dual agonist + lifestyle | 40–52 weeks | −11–15% | −16–20% | −4–5% | Comparable lean/fat ratio improvement to exercise-based loss |
| Exercise-only meta-analyses (WHO, EASO, 2022–2024) | Aerobic + resistance exercise | 12–24 weeks | −3–6% | −5–7% | +1–2% or stable | Improves VO₂max, insulin sensitivity, muscle function; crucial for lean mass preservation |

**Supplementary Table S1.** Summary of the major studies on GLP-1RAs and exercise interventions
